# Characterization of Choroidal Morphology and Vasculature in the Phenotype of Pachychoroid Diseases by Swept-Source OCT and OCTA

**DOI:** 10.3390/jcm11113243

**Published:** 2022-06-06

**Authors:** Bingjie Qiu, Xinyuan Zhang, Zhiqing Li, Jay Chhablani, Hao Fan, Yanhong Wang, Rui Xie

**Affiliations:** 1 Beijing Tongren Eye Center, Beijing Tongren Hospital, Capital Medical University, Beijing 100730, China; qiubingjie16@mail.ccmu.edu.cn (B.Q.); sherry1996@ccmu.edu.cn (R.X.); 2 Beijing Retinal and Choroidal Vascular Disorders Study Group, Beijing 100730, China; 3 Tianjin Medical University Eye Hospital, Tianjin Medical University, Tianjin 300392, China; drzhiqing_li@163.com (Z.L.); fh960316@163.com (H.F.); 4 UPMC Eye Center, University of Pittsburgh, Pittsburgh, PA 15213, USA; jay.chhablani@gmail.com; 5 Department of Epidemiology and Biostatistics, Institute of Basic Medical Sciences Chinese Academy of Medical Sciences & School of Basic Medicine Peking Union Medical College, Beijing 100005, China; wyhong826@pumc.edu.cn

**Keywords:** pachychoroid disorders, polypoidal choroidal vasculopathy, neovascular age-related macular degeneration, central serous chorioretinopathy, swept-source OCT, OCTA quantitative analysis

## Abstract

The objective of this study was to characterize the choroidal morphology and vasculature in pachychoroid diseases (PCD). A total of 49 eyes with polypoidal choroidal vasculopathy (PCV), 43 eyes with neovascular age-related macular degeneration (nAMD), and 50 eyes with central serous chorioretinopathy (CSC), along with 80 healthy eyes, were enrolled in this nested case-control study. The swept-source optical coherent tomography (OCT), OCT angiography, and En face images were quantitatively analyzed. Multivariate logistic regression models showed that older age and increased vessel density (VD) in the choriocapillaris (CC) layer were independent risk factors for both PCV (*p*_age_ < 0.001, *p*_VD_ = 0.004), and nAMD (*p*_age_ < 0.001, *p*_VD_ = 0.005). Decreased VD in the Sattler’s layer was an independent risk factor for PCV (*p* = 0.014). Increased VD in the Haller’s layer was an independent risk factor for CSC (*p* = 0.001). The proportion of the diffuse type of collateral circulation in the Sattler’ layer in CSC group was significantly higher than in the other three groups (*p* < 0.001). We concluded that the involvement of the blood flow in the CC, Haller’s, and Sattler’s layers are differently affected in CSC, nAMD, and PCV eyes, indicating the different pathological mechanism underlying the phenotype of PCD. The age-dependent establishment of collateral circulation in the Sattler’s layer may play a compensatory role regarding ischemic injury in the development of PCD.

## 1. Introduction

Pachychoroid diseases (PCD), first proposed by Warrow et al. in 2013, are characterized by morphological changes and dysfunction of the choroid [1]. Central serous chorioretinopathy (CSC) and polypoidal choroidal vasculopathy (PCV) are the two predominant phenotypes of PCD. Several studies have shown CSC has complicated interleaving effect with PCV; about 36% of chronic CSC with type I macular neovascularization (MNV) will eventually progress to PCV [2]. Furthermore, the genetic locus underlying a phenotypic difference between PCV and neovascular age-related macular degeneration (nAMD) is also shown, although PCV has long been considered to be a subtype of nAMD [3,4]. Controversially, Asian patients with nAMD are frequently found to be lacking soft drusen, sharing some typical characteristics resembling PCD, such as choroidal hyperpermeability and thickening, suggesting that an overlap in pathogenesis may exist between nAMD and PCV [5,6]. Therefore, the pathogenesis of PCV, CSC, and nAMD and their relationship remain a significant challenge and warrant further investigation.

PCD is a group choroidal origin disorders; in-depth understanding of the anatomical features can help to further excavate the pathogenesis of PCD. Several studies have shown that in PCD, the location of pathologically dilated Haller vessels correlated with zones of reduced choriocapillaris (CC) flow, and collateral circulation plays an important role in protecting the retina from ischemic injury [7,8]. Although clinical manifestations of PCD vary considerably, tremendous development of fundus imaging technologies, including swept-source optical coherence tomography (SS-OCT), offer a pathological view, providing more details of vasculature in both the retina and the choroid. The new development of OCT angiography (OCTA) also provides detailed information of the choroid blood flow and three-dimension visualization of micro vessels, recognizing the choroid stratification, quantitatively analyzing vessel density and collateral circulation [9,10,11].

In this study, we tested the hypothesis that the blood flow is differentially regulated in the choroidal layers in different phenotypes of PCD, and that the morphological changes of the choroidal layers reflect their genetic variation and their pathogenesis. Furthermore, we also tested the hypothesis that age-dependent establishment of collateral circulation in the Sattler’s layer may explain the intricate pathological relationship between CSC, PCV, and nAMD.

## 2. Materials and Methods

### 2.1. Subjects

Subjects with PCV, nAMD, and CSC, as well as normal subjects, matched for gender and refractive error between September 2019 to September 2021, were enrolled in this case-control study at Beijing Tongren Hospital. This study was approved by the Ethics Committee of Beijing Tongren Hospital, Capital Medical University, and adhered to the tenets of the Declaration of Helsinki. All the subjects signed the informed consent form before participation.

Inclusion criteria: naïve eyes with PCV, nAMD, and CSC, refractive error ≤6.0 diopter were clinically diagnosed by the fundus examination, fundus color photography, SS-OCT, and indocyanine green angiography (ICGA), according to EVEREST study report 2, modified EVEREST criteria [12,13], and the 2020 Asia-Pacific Eye Image Association PCV expert consensus of the working group [14]. CSC was diagnosed then diagnosed. 

Exclusion criteria included: (1) complications from other fundus diseases, such as macular hole, diabetic retinopathy, and pathological myopia, (2) macular edema secondary to retinal vascular diseases, (3) choroidal tumors, including metastatic carcinoma, melanoma, and others, (4) previous vitreoretinal surgery, (5) serious systemic organic diseases or inability to tolerate examination, (6) phakia or intraocular lens eyes, (7) serious anterior segment disorders, such as cataracts and keratopathy, affecting the fundus image quality; OCT Scans with low quality(<40/100) were excluded from the study [15].

### 2.2. Determination of the Cutoff Value Range of the Pachychoroid Diseases

Subjects were stratified by age (beginning at age 20 years), and the positive likelihood ratio of patients with normal eyes and PCD patients was calculated according to per 100 μm/50 μm/25 μm subfoveal choroidal thickness (SFCT). The positive likelihood ratio is close to 1; the corresponding range of SFCT is the diagnostic threshold to distinguish normal eyes from PCD.

The following value range was determined by the likelihood ratio test: among the 20–39-year-olds, the threshold of the diagnostic value range of PCD was 320–330 μm [likelihood ratio (LR): 1.17]. Among the 40–59-year-olds, the range of PCD was 330–340 μm (LR: 1.07). Among the 60–79-year-old, the threshold range was 250–275 μm (LR: 1.07). The threshold diagnostic value of PCD was 200–225 μm (LR: 1.00) for the ≥ 80-year-olds.

### 2.3. Eye Examination

All included subjects underwent comprehensive ocular examinations, including the following tests: best-corrected visual acuity (BCVA), non-contact intraocular pressure (TX20 Automatic Non-contact Tonometer, Canon Co., Ltd., Tokyo, Japan), slit-lamp microscopic examination (SL-IE Slit Lamp Microscope, Topcon Co., Ltd., Tokyo, Japan), color fundus photography (CR-1 non-mydriatic Fundus Camera, Canon Co., Ltd.) with mydriasis, optical coherence tomography (OCT), fluorescein angiography (FA), and indocyanine green angiography (ICGA).

### 2.4. Examination by Swept-Source Optical Coherence Tomography

#### 2.4.1. OCT B-Scan Images Acquisition

All enrolled subjects were examined by SS-OCT (DRI Triton, Topcon, Tokyo, Japan) with mydriasis from 13 PM to 16 PM. A total of 12 high-resolution B-scan images, which passed through the fovea, were obtained through the macular area radial scanning mode with the scanning range of 9 × 9 mm.

#### 2.4.2. Measurement of Choroidal Thickness

Choroidal thickness (ChT) refers to the distance between the high reflection zone of the Bruch’s membrane under RPE and the outer choroidoscleral boundary. The boundary of the Sattler’s and Haller’s layers was determined manually, according to the morphological magnifications of the choroidal vasculature (including the size of the vessel lumen) [16]. ChT in nine macular regions was measured automatically using TOPCON Advanced Boundary Segmentation-TABS software. The Early Treatment Diabetic Retinopathy Study Classification (ETDRS) regions are divided into nine parts: one 1 mm diameter circle at the center, four inner-quarter annuluses between the 1 and 3 mm diameter circles on the outside, and four outer quarter annuluses between the 3 and 6 mm diameter circles. Two independent ophthalmologists completed the entire measurement process. To correct the thickness measurements in cases of errors, computer-aided manual correction of OCT segmentation was applied.

### 2.5. Images Acquisition of SS-OCTA and En Face SS-OCT

OCTA and En face images were acquired using the IMAGEnet^®^ 6 software (Version 1.22.13993,Topcon, Tokyo, Japan) at 6 mm x 6 mm scanning mode. Individual images of the choriocapillaris, Haller’s, and Sattler’s plexus were generated by Topcon IMAGEnet^®^ 6 software, with computer-aided manual correction. We manually moved down the segmentation lines to define the CC, Sattler’s, and Haller’s layers, according to the morphological characters of the choroidal vasculature as we described above.

The choriocapillaris layer shows better visualizations in SS-OCTA, while in the outer choroid, SS-OCTA cannot capture the choroidal vasculature as fully as En face SS-OCT, leading to the consistent underestimation of choroid vascular density (CVD) in the outer choroid vessels in comparison with SS-OCT. This is likely due to a very low signal-to-noise ratio in the outer choroid vessels, because of the sensitivity roll-off with increasing imaging depth, leading to fewer signals meeting the decorrelation threshold on SS-OCTA [17]. For those reasons, we used OCTA images to quantify blood flow density in the choriocapillaris layer and En face images in the Sattler’s and Haller’s layers for CVD [17].

#### 2.5.1. Vascular Density in Choriocapillaris and CVD in Sattler’s and Haller’s Layers

**Figure 1:**
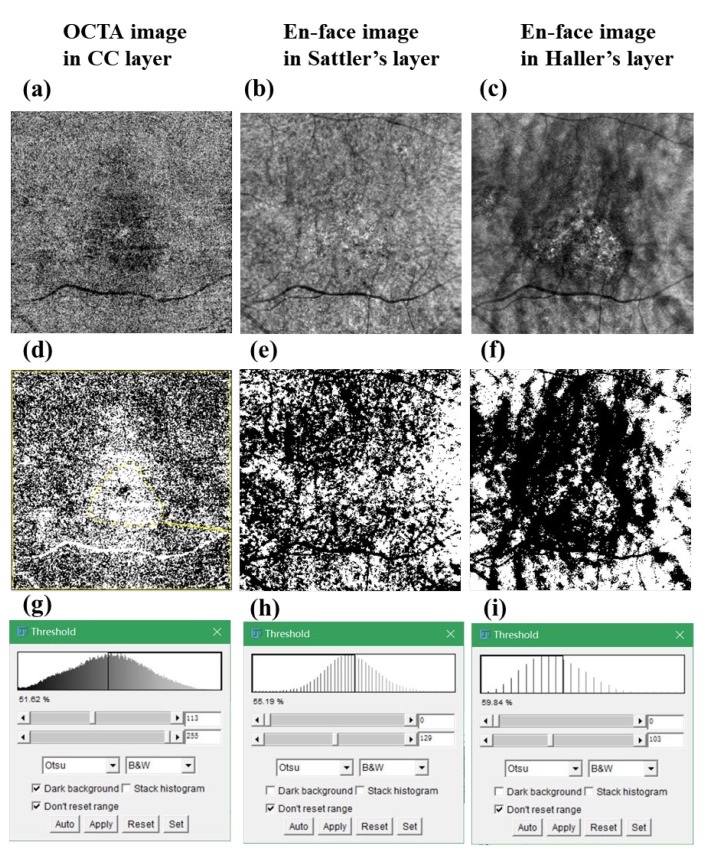
Representative images of an eye with CSC to describe the process for the determination of vessel density in different choroid layers using image J. (**a**–**c**) OCTA image in CC layer (**a**), En face images in Sattler’s (**b**), and Haller’s layers (**c**), respectively. (**d**–**f**) Binarized images corresponding with (**a**–**c**), (**g**–**i**); the VD was automatically calculated by image J software with the Otsu method. CC: choriocapillaris; VD: vessel density.

Image J software (version 1.49; National Institutes of Health, Bethesda, MD, USA) was utilized to quantified choroidal blood flow in the CC layer and CVD in the Sattler’s and Haller’s layers. OCTA images or En face images were reverted into the 8-bit integer data type and binarized using the image J Otsu’s method [18] (Figure 1). The percentage of white or black pixels, the percentage of choriocapillaris (white), and choroidal vessels (black) were automatically calculated.

#### 2.5.2. Semi-Quantification of the Collateral Circulation

**Figure 2:**
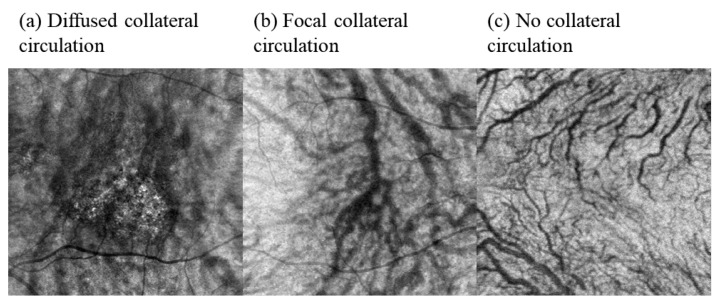
Representative 6 × 6 mm En face images showing the diffused collateral circulation (**a**), focal collateral circulation (**b**) and no collateral circulation vessels (**c**).

In this study, a semi-quantitative method was applied to describe the existence of collateral circulation by combining the En face and ICGA images. Two independent ophthalmologists ascertained the semi-quantification results. The upper and lower vortex veins anastomosed, and the disappearance of a watershed indicated the existence of collateral circulation [8]. According to the distribution of collateral circulation, it can be divided into: (1) diffuse collateral circulation—the proportion of collateral circulation ≥ two quadrants; (2) local collateral circulation—the proportion of collateral circulation < two quadrants in images; (3) no collateral circulation (Figure 2a–c).

### 2.6. Sample Size Determination

The final sample size was determined by the Power Analysis and Sample Size software (PASS 2022, NCSS LLC, Kaysville, UT, USA), based on our pilot study result. The minimum number per arm (sample size) was 13 eyes to detect the difference between the four groups with the designed power (1 − beta = 90%) at 95% confidence level (alpha = 0.05), as we previously described [19]. We increased the sample size to above 35 eyes in each group, considering the variability (even in normal controls) in the SFCT or VD values.

### 2.7. Statistical Analysis

SPSS software (SPSS, Inc. 25.0, Chicago, IL, USA) was applied for statistical analysis. Baseline parameters, including central macular choroidal thickness and vessel density in the CC, Sattler’s, and Haller’s layers, were described by means ± standard deviation (mean ± SD) or median (interquartile range, IQR). The comparisons among groups were analyzed by one-way analysis of variance (ANOVA) or the Kruskal–Wallis test, according to the data distribution. Multivariate logistic regression models were utilized for controlling the age, gender, and refractive errors to investigate the associations between the different phenotypes of PCV. The odds ratio (OR) for group comparison was analyzed using the normal control as the reference, OR > 1 means the variable is an independent risk factor for the study group; OR < 1 means the variable is a protective factor for the disease. When the disease was used as the reference, an OR > 1 means the higher level of the independent variable was in the study group, whereas an OR < 1 means the higher level of the independent variable was in the reference group. Statistical significance was defined as *p* < 0.05.

## 3. Results

### 3.1. Clinical Characteristics and Baseline Demographics of the Study Subjects

A total of 49 eyes with PCV, 43 eyes with nAMD, and 50 eyes with CSC were enrolled in this nested case-control study; 80 normal eyes matched for sex and refractive error were included as the normal control. The onset age of CSC patients is younger than that in PCV and nAMD groups, and age was statistically different among the four groups (*p_age_* < 0.001). There was no significant difference in gender and refraction among PCV, nAMD, CSC and control groups (*g_ender_ =* 0.954, *r_efractive_ =* 0.083, respectively) (Table 1).

### 3.2. Comparison of SFCT in Normal Control, PCV, nAMD, and CSC

**Figure 3:**
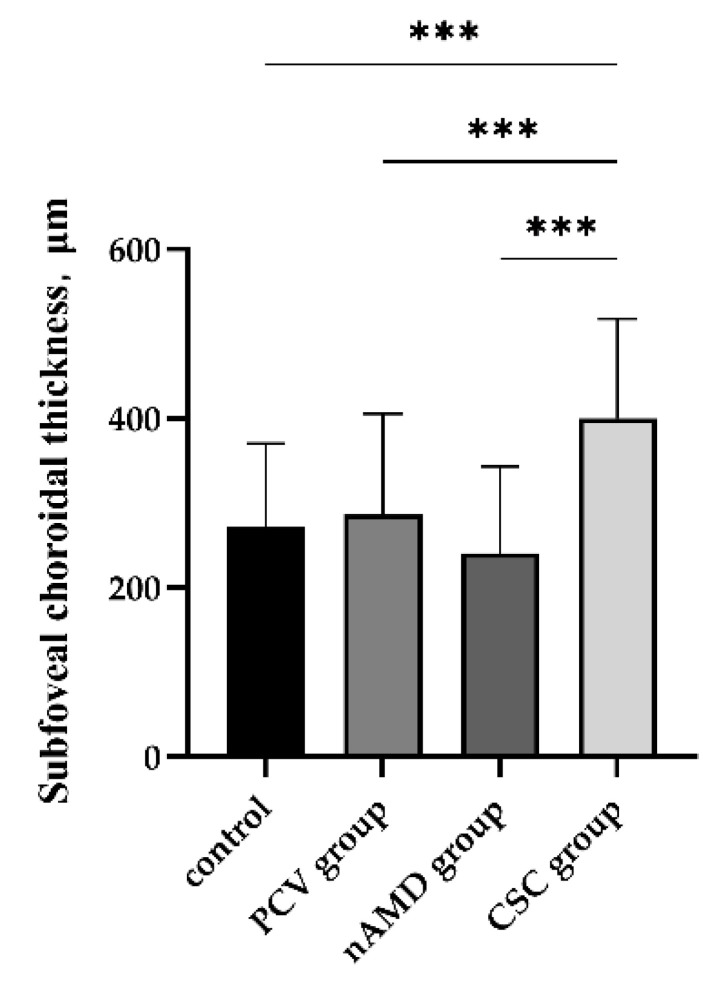
Comparison of the SFCT between the PCV, nAMD, and CSC groups and the normal control group. PCV: polypoidal choroidal vasculopathy; nAMD: neovascular age-related macular degeneration; CSC: central serous chorioretinopathy. *** *p* < 0.001.

There were statistical differences in SFCT among the PCV, CSC, nAMD, and normal groups (*p _all_* < 0.001); the SFCT in the CSC group was statistically thicker than that in normal control (*p _CSC_* _vs._ *_control_*< 0.001), PCV (*p _CSC_* _vs._ *_PCV_*< 0.001), and nAMD (*p _CSC_* _vs._ *_nAMD_*< 0.001) groups (Figure 3, Table 2). Multivariate logistic regression analysis showed that age (OR = 1.09, 95% CI 1.05–1.14, *p* < 0.001) was older and SFCT (OR = 1.52, 95% CI 1.01–2.30, *p* = 0.045) was significantly thicker in PCV in comparison with the normal control. Age in the nAMD group was older in comparison with normal control group (OR = 1.07, 95% CI 1.03–1.11, *p* = 0.001). Furthermore, age was younger in the CSC group compared with that in normal control, PCV, and nAMD groups, respectively (*p _CSC_* _vs._ *_normal_*= 0.013; *p _CSC_* _vs._ *_PCV_* < 0.001; *p _CSC_* _vs._ *_nAMD_* = 0.007). SFCT was statistically thicker in the CSC group compared with that in normal control and nAMD (OR = 3.48, 95% CI 2.01–6.02, *p _CSC_* _vs._ *_normal_* < 0.001; OR = 8.16, 95% CI 1.30–51.14, *p _CSC_* _vs._ *_nAMD_* = 0.025, respectively). SFCT was significantly thinner in nAMD eyes than that in PCV eyes (OR = 3.48, 95% CI 2.01–6.02, *p* < 0.001). Moreover, compared to nAMD groups, the number of males was significantly higher in the CSC groups (*p* = 0.029) (Table 3).

Eyes with pachychoroid (thickened choroid) in CSC, PCV, and nAMD were 80%, 48.98%, and 23.26%, respectively, according to the criteria described above.

### 3.3. Comparison of Blood Flow or Vessel Density in Choroidal Layers between the PCV, nAMD, CSC, and Normal Control Groups

There was no statistical difference in VD in the CC layer between the CSC, PCV, nAMD, and healthy control groups by Kruskal–Wallis analysis (*p _all_* = 0.103) (Table 2). Multivariate logistic regression analysis showed age (OR = 1.10, 95% CI 1.06–1.15, *p* < 0.001) and VD in the CC layer (OR = 1.47, 95% CI 1.13–1.91, *p* = 0.004) were independent risk factors for PCV eyes in comparison with the normal control. Similarity, age (OR = 1.11, 95% CI 1.06–1.16, *p* < 0.001) and VD in the CC layer (OR = 1.61, 95% CI 1.16–2.30, *p* = 0.005) were independent risk factors for nAMD eyes when compared with the normal control. Age was younger for CSC subjects in comparison with the normal control (OR = 0.88, 95% CI 0.91–0.98, *p* = 0.002), with the PCV eyes (OR = 0.76, 95% CI 0.67–0.85, *p <* 0.001), and with the nAMD eyes (OR = 0.70, 95% CI 0.58–0.85, *p <* 0.001) (Table 3).

There was no statistical difference in VD in the Sattler’s layer among the four groups by Kruskal–Wallis analysis (*p _all_* = 0.151) (Table 2). Multiple variable logistic regression analysis showed that age (OR = 1.09, 95% CI 1.04–1.13, *p* < 0.001) and VD in the Sattler’s layer (significantly decreased, OR = 0.86, 95% CI 0.76–0.97, *p* = 0.014) were independent risk factors for PCV eyes in comparison with normal control eyes. Age was an independent risk factor for nAMD (*p* = 0.001) and CSC eyes (*p* < 0.001) compared with normal control eyes. Furthermore, age was younger in CSC subjects than PCV (*p* < 0.001) and nAMD subjects *(p* < 0.001) (Table 3).

There was statistical difference for VD in the Haller’s layer between the four groups using the ANOVA test (*p _all_* < 0.001, *p _CSC_* _vs._ *_control_*< 0.001, *p _CSC_* _vs._ *_PCV_*< 0.001, *p _CSC_* _vs._ *_nAMD_* < 0.001) (Table 2). The multivariable logistic regression model showed age was an independent risk factors for PCV (*p* < 0.001) and nAMD subjects (*p* <0.001) in comparison with the normal control group. Younger age (*p* = 0.002) and increased VD in the Haller’s layer (*p* = 0.001) were independent risk factors for CSC eyes when compared with normal control eyes after controlling for gender and refractive error. CSC subjects were significantly younger than PCV (*p* < 0.001) and nAMD subjects (*p* = 0.010). Furthermore, the number of males was significantly higher (*p* = 0.046) and VD in the Haller’s layer was decreased (OR = 1.34, 95% CI 1.06–1.70, *p* = 0.015) in CSC eyes compared with nAMD eyes (Table 3).

### 3.4. Comparison of the Proportion of Collateral Circulation between the PCV, nAMD, CSC, and Normal Control Groups

**Figure 4:**
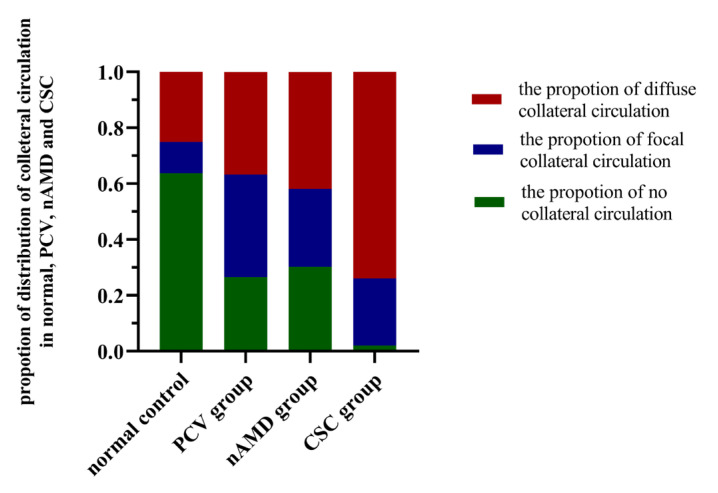
Comparisons of the proportion of the diffuse type (red column), focal type (blue column) and “no” type (green column) of collateral circulation among the four groups. There was a statistical difference in the proportion of diffuse collateral circulation between the PCV, nAMD, CSC, and normal control groups (χ^2^ = 31.09, *p* < 0.001). PCV: polypoidal choroidal vasculopathy; nAMD: neovascular age-related macular degeneration; CSC: central serous chorioretinopathy; Pachy-PCV: PCV with pachychoroid.

In the normal eyes, the proportion of diffuse collateral circulation, focal collateral circulation, and no collateral circulation was 25.00%, 11.25%, and 63.75%, respectively. In eyes with PCV, the proportion was 36.73%, 36.73%, and 26.53%, respectively. In eyes with nAMD, the proportion was 41.86%, 27.91%, and 30.23%, while in eyes with CSC, the proportion was 74.00%, 24.00%, and 2.00%, respectively. There was a statistical difference in the proportion of diffuse collateral circulation between the PCV, nAMD, CSC, and normal control groups (χ^2^ = 31.09, *p* < 0.001); the CSC group (74.00%) showed a higher proportion of diffuse collateral circulation than the normal eye (*p* < 0.05), PCV (*p* < 0.05), and nAMD (*p* < 0.05) groups. Besides, there were also statistical differences in the proportion of focal collateral circulation between the PCV, nAMD, CSC, and normal control groups (χ^2^ = 12.08, *p* = 0.007). The PCV group (36.73 %, *p* < 0.05) showed a higher proportion of focal collateral circulation than the normal control group (*p* < 0.05). Finally, there were still statistical differences in the proportion of no collateral circulation between the PCV, nAMD, CSC, and normal control groups (χ^2^ = 54.88, *p* < 0.001), and the normal control group showed more frequent no collateral circulation than the PCV (*p* < 0.05), nAMD (*p* < 0.05), and CSC (*p* < 0.05) groups, and the CSC group showed less frequent no collateral circulation than the PCV (*p* < 0.05) and nAMD (*p* < 0.05) groups (Figure 4).

## 4. Discussion

In this study, multivariate logistic regression models showed that SFCT in CSC and PCV eyes was significantly thicker than that in normal healthy and nAMD eyes. Older age and increased VD in the CC layer were independent risk factors for both PCV and nAMD. Decreased VD in the Sattler’s layer was an independent risk factor for PCV eyes. Furthermore, VD in the Haller’s layer in eyes with CSC was significantly higher than that in the normal control and nAMD groups. These results confirmed that choroidal thickness is a good imaging marker for CSC, PCV, and nAMD; the involvement of the blood flow in the Haller’s and Sattler’s layers is differently affected in CSC, nAMD, and PCV eyes, indicating the different pathological mechanism behind each disease. The proportion of the diffuse type of collateral circulation was significantly different between the CSC, PCV, nAMD, and normal control eyes (CSC > PCV > nAMD > normal control), indicating that the age-dependent establishment of collateral circulation in the Sattler’s layer may play a compensatory role in ischemic injury in the development of PCD.

After adjusting for age, gender, and refractive error, higher VD in the choriocapillaris was found in both the PCV and nAMD groups compared with the normal control group, indicating that ischemia in PCV and nAMD, but not CSC, may contribute to the congestion of the vessels in the choriocapillaris, which is consistent with previous studies [18,20]. This result further confirmed that ischemia primarily contributed to the pathogenesis of PCV and nAMD.

The development of collateral circulation is one of the major mechanisms in response to ischemia in different tissues, including heart, brain, and eye tissues [21]. When ischemic injury occurs, the choroidal collateral circulation is critically important as an adaption of the eye to prevent the damage from ischemic insults, playing a compensatory role in re-establishing blood perfusion under fluid shear stress (FSS) and inflammation [21,22], providing a network of specialized endogenous bypass vessels in the choroid [7,21,22]. Several imaging studies have shown that either normal or pachychoroidal eyes exhibit choroidal vasculature remodeling at the posterior [7,8]. In this study, arteriovenous anastomosis was found to be randomly distributed in the choroidal, Sattler’s, and Haller’s layers in eyes with CSC, PCV, and nAMD, but the percentage of the diffused collateral circulation was significantly higher in CSC (with younger age) than that in the PCV and nAMD groups; this can explain why decreased VD in the Sattler’s layer was found in PCV and nAMD eyes, but not in CSC eyes. It also further confirmed that the development of collateral circulation is age-dependent [8].

Low blood oxygen content in collateral vessels further leads to the proliferative senescence and subsequent apoptosis of vascular endothelial and smooth muscle cells in collateral vessels [23]; this can explain why 36% of chronic CSC will eventually be complicated with type 1 CNV [2], with an outcome of PCV in many instances (such as pachdrusen) [24]. The highest proportion of the diffused type of collateral circulation in CSC in our study promoted the investigation of whether CSC is an early stage of PCV; we hypothesized that CSC could eventually progress to PCV when the ability to establish collateral circulation decreases and can no longer compensate for the ischemic state.

PCV has been classified into different subtypes using ICGA. In 2013, Kawamura et al. [25] proposed two distinguishable subtypes of PCV according to different filling patterns on ICGA: type 1 PCV presents a feeder and a draining vessel, with small non-pulsating polys and thinner choroidal thickness; type 2 PCV presents larger and pulsating polyp lesions, reticular vessels, and localized choroidal vessel dilation. Tan et al. further refined this classification to types A, B, and C of PCV [26]. In this study, we simply categorized the phenotypes of PCV and nAMD by choroidal thickness measured by OCT; the invasive method provides new insight towards deeper understanding of the pathogenesis of PCD and the intricate relationship between CSC, PCV, and nAMD.

The percentage of the thickened choroid (pachychoroid) in CSC, PCV, and nAMD eyes was 80%, 48.98%, and 23.26%, respectively; in this study, almost half of PCV and 1/4 of nAMD eyes were found to be pachychoroid, reflecting the different pathological mechanisms between PCV and nAMD. Furthermore, a significant difference in the percentage of eyes with pachychoroid between the three groups (CSC >PCV> nAMD) can also explain why SFCT was significantly higher in the CSC and PCV groups (with a higher proportion of eyes with pachychoroid in the two groups). In the Haller’s layer, VD was higher in PCV eyes than that in normal control, but no statistically significant difference was found, possibly due to the fact that non-pachychoroid eyes do not exhibit enlarged vessels, as reported previously [3]; the non-pachychoroid PCV eyes, which occupied approximately 50% of the PCV group, may offset the result. This warranted the further investigation of the imaging characteristics of the subtypes of PCV and nAMD. The different imaging magnifications from this study also challenge the traditional concept that PCV is a subtype of nAMD.

In previous a study, a branching neovascular network in PCV was found to be a subretinal neovascularization, located under the RPE and between Bruch’s membrane (type 1 macular neovascularization, exhibited in above 90% of PCV), which can be predominately distinguished by SS-OCT. Furthermore, the double-layer sign, a sign of type 1 CNV, has been reported to be the pachychoroid phenotype [27]. This phenotype is similar to that in the non-pachychoroid nAMD eyes, which was found to be present in type 1 CNV. On the other hand, in this study, almost 100% of non-pachychoroid PCV was found to be “naked-PCV” by SS-OCT B scan, as was described by Dansingani et al. in 2018 [28]. This phenotype is similar to the type 2 MNV nAMD. In addition, it was found that non-pachychoroid PCV resembled typical AMD in terms of ChT, choroid vessel area, and distribution characteristics of the pachyvessels [3,4].

Increasing genetic evidence has been shown to contribute to the pathogenesis of PCD. Lehmann et al. [29] found that a thick choroid presented in 50% of the CSCR relatives, indicating that the pachychoroidal trait may potentially be a dominantly inherited condition; both exogenous and endogenous factors synergistically promote the occurrence and development of PCD. Recent studies have suggested similarities in clinical phenotypes and genotypes of PCV, but differences in drusen-driven AMD [30]. Hence, we deem that the pathogenesis of PCV with pachychoroid and nAMD with pachychoroid, sharing similar clinical phenotypes, may overlap, but further study is required to validate this conclusion and to investigate the intricate, yet common relationship between PCV and nAMD.

Although more intensive studies have focused on choroidal thickness in recent years, until now, there has been no unified international protocol for the measurement of the choroidal thickness. In this study, ChT in nine macular regions was measured automatically using TOPCON Advanced Boundary Segmentation-TABS software, followed by the computer-aided manual correction of OCT segmentation to minimize the subjective errors [19,31]. Furthermore, we applied SS-OCT for image acquisition, the most ideal method for obtaining SFCT due to its high resolution of choroid and sclera currently with the scanning depth of 2.6 mm, 100,000 scans per second, and 8 μm axial resolution of A-scan.

Finally, the signal intensity within the choroid may be influenced by hematologic and pathological changes, such as changes in subretinal fluid and retinal pigment epithelia detachment, especially in PCV and CSC eyes, where optimization algorithms remain challenging [4].

There are still some limitations in this study. Although we used a healthy normal cohort as the control to reduce the heterogeneity caused by the quantity imbalance, the sample size of the study group is still limited, and longitudinal morphological observation of the choroid is warranted. Moreover, although projection artefact removal was used during OCT scanning, the shadows from the superficial plexus projection tails affects the quantitative assessment; therefore, projection artifacts of VD should be considered [32]. Furthermore, as one of the objective biomarkers for evaluating the choroid, SFCT depends on variable physiological and pathological factors of the body, including age, refractive error, axial length or diurnal changes, and individual differences [7,8,11,12,13]. Studies in Korea [33] and Japan [34] confirmed by multiple linear regression analysis showed that age is the most important factor affecting choroidal thickness, followed by diopter. Other variable factor will be further investigated in the future studies.

## 5. Conclusions

In summary, The Choroidal morphology and vasculature of the phenotypes of PCD were differentially regulated, which implied the different pathological mechanisms underlying PCV, nAMD, and CSC. Age-dependent establishment of collateral circulation in the Sattler’s layer may play a compensatory role regarding ischemic injury in the development of PCD.

## Figures and Tables

**Table 1:** The demographic characteristics of all the enrolled subjects.

	Healthy Control Subjects (Eyes) 43(80)	PCV Subjects (Eyes) 49(49)	nAMD Subjects (Eyes) 36(43)	CSC Subjects (Eyes) 45(50)	*F/χ^2^*	*p* Value
Age, y (mean ± SD)	53.60 ± 15.97	67.44 ± 9.47	67.83 ± 7.85	48.53 ± 9.74	60.48 ^a^	<0.001 *
Gender, male/female (N)	31/12	29/14	24/12	31/14	0.33 ^b^	0.954
Refractive, D (eyes)	−0.58 ± 1.53	−0.22 ± 1.44	−0.21 ± 1.60	−0.33 ± 0.99	6.67 ^a^	0.083

* Statistically significant: *p* ≤ 0.05; ^a^ Kruskal–Wallis; ^b^ Chi-square test. SD: standard deviation; D: diopter; PCV: polypoidal choroidal vasculopathy; nAMD: neovascular age-related macular degeneration; CSC: central serous chorioretinopathy. N: number.

**Table 2:** Single factor comparisons of SFCT and the vessel density in the CC, Sattler’s, and Haller’s layers between the PCV, nAMD, CSC, and normal control groups.

	Control (N = 43)	PCV (N = 43)	nAMD (N = 36)	CSC (N = 45)	*F/χ^2^*	*p* Value
SFCT, μm (mean ± SD)	270.58 ± 99.56	285.73 ± 119.28	243.51 ± 102.12	399.92 ± 117.15	43.60 ^b^	<0.001 *
VD in CC layer, % (mean ± SD)	49.14 ± 1.37	49.60 ± 2.33	49.37 ± 2.47	49.91 ± 1.47	6.18 ^b^	0.103
VD in Sattler’s layer, % (mean ± SD)	53.47 ± 3.51	52.04 ± 3.63	52.02 ± 5.52	52.91 ± 3.06	5.30 ^b^	0.151
VD in Haller’s layer, % (mean ± SD)	50.61 ± 5.12	50.56 ± 7.05	50.35 ± 5.85	55.60 ± 6.60	9.13 ^a^	< 0.001 *

* Statistically significant: *p* < 0.05, according to the type of data and the data distribution. ^a^ one-way ANOVA analysis, post-hoc Bonferroni’s statistic; ^b^ Kruskal–Wallis analysis. VD: vessel density; ‘SD: standard deviation; PCV: polypoidal choroidal vasculopathy; nAMD: neovascular age-related macular degeneration; CSC: central serous chorioretinopathy.

**Table 3:** Multivariate logistic regression models showing the statistical difference in SFCT and the vessel density in the CC, Sattler’s, and Haller’s layers between the PCV, nAMD, CSC, and normal control groups.

SFCT				VD in CC Layer				VD in Sattler’s Layer				VD in Haller’s Layer			
Models/Factors	OR	95% CI	*p* Value	Factors	OR	95% CI	*p* Value	Factors	OR	95% CI	*p* Value	Factors	OR	95% CI	*p* Value
**PCV vs. Control**															
Gender (Ref: male)	0.94	0.40–2.21	0.885	Gender (Ref: male)	1.04	0.42–2.57	0.926	Gender (Ref: male)	0.69	0.27–1.75	0.435	Gender (Ref: male)	0.99	0.40–2.44	0.984
Age	1.09	1.05–1.14	<0.001 *	Age	1.10	1.06–1.15	<0.001 *	Age	1.09	1.04–1.13	<0.001 *	Age	1.08	1.04–1.12	<0.001 *
Refractive	0.99	0.74–1.34	0.959	Refractive	1.06	0.79–1.42	0.716	Refractive	1.16	0.81–1.66	0.418	Refractive	1.21	0.87–1.68	0.269
SFCT (per 100μm)	1.52	1.01–2.30	0.045 *	VD in CC layer	1.47	1.13–1.91	0.004 *	VD in Sattler’s layer	0.86	0.76–0.97	0.014 *	VD in Haller’s layer	1.03	0.96–1.11	0.418
**nAMD vs. Control**															
Gender (Ref: male)	0.74	0.27–2.03	0.561	Gender (Ref: male)	1.15	0.42–3.14	0.781	Gender (Ref: male)	1.16	0.44–3.07	0.771	Gender (Ref: male)	0.97	0.38–2.51	0.950
Age	1.07	1.03–1.11	0.001 *	Age	1.11	1.06–1.16	<0.001 *	Age	1.08	1.03–1.13	0.001 *	Age	1.08	1.03–1.12	<0.001 *
Refractive	1.11	0.81–1.51	0.527	Refractive	1.11	0.80–1.54	0.535	Refractive	1.03	0.74–1.44	0.866	Refractive	1.14	0.83–1.58	0.423
SFCT (per 100μm)	0.79	0.48–1.31	0.360	VD in CC layer	1.61	1.16–2.3	0.005 *	VD in Sattler’s layer	0.94	0.82–1.07	0.322	VD in Haller’s layer	1.00	0.92–1.09	0.998
**CSC vs. Control**															
Gender (Ref: male)	1.25	0.45–3.46	0.673	Gender (Ref: male)	0.88	0.36–2.11	0.767	Gender (Ref: male)	0.90	0.37–2.18	0.812	Gender (Ref: male)	1.37	0.53–3.55	0.521
Age	0.95	0.91–0.99	0.013 *	Age	0.88	0.91–0.98	0.002 *	Age	0.94	0.91–0.97	<0.001 *	Age	0.94	0.91–0.98	0.002 *
Refractive	1.10	0.73–1.66	0.637	Refractive	1.30	0.92–1.82	0.132	Refractive	1.32	0.94–1.87	0.111	Refractive	1.24	0.85–1.81	0.265
SFCT (per 100μm)	3.48	2.01–6.02	<0.001 *	VD in CC layer	1.19	0.89–1.59	0.243	VD in Sattler’s layer	0.98	0.87–1.11	0.780	VD in Haller’s layer	1.15	1.06–1.25	0.001 *
**nAMD vs. PCV**															
Gender (Ref: male)	0.87	0.31–2.42	0.784	Gender (Ref: male)	1.68	0.58–4.86	0.342	Gender (Ref: male)	1.63	0.57–4.68	0.360	Gender (Ref: male)	1.96	0.61–6.36	0.262
Age	0.97	0.92–1.02	0.258	Age	0.97	0.91–1.03	0.324	Age	0.99	0.94–1.05	0.814	Age	0.97	0.91–1.04	0.377
Refractive	1.15	0.83–1.59	0.409	Refractive	0.85	0.60–1.20	0.353	Refractive	0.91	0.62–1.32	0.602	Refractive	0.77	0.50–1.19	0.235
SFCT (per 100μm)	0.53	0.32–0.87	0.013 *	VD in CC layer, %	1.04	0.83–1.30	0.738	VD in Sattler’s layer	1.05	0.92–1.20	0.494	VD in Haller’s layer	0.99	0.91–1.08	0.819
**CSC vs. PCV**															
Gender (Ref: male)	1.48	0.31–7.14	0.623	Gender (Ref: male)	1.11	0.22–5.53	0.903	Gender (Ref: male)	2.43	0.38–15.67	0.351	Gender (Ref: male)	2.09	0.33–13.22	0.432
Age	0.77	0.68–0.86	<0.001 *	Age	0.76	0.67–0.85	<0.001 *	Age	0.72	0.62–0.84	<0.001 *	Age	0.75	0.66–0.85	<0.001 *
Refractive	0.82	0.44–1.53	0.525	Refractive	0.78	0.41–1.4	0.447	Refractive	0.63	0.21–1.87	0.400	Refractive	0.56	0.24–1.27	0.163
SFCT (per 100μm)	1.37	0.68–2.77	0.385	VD in CC layer	0.82	0.52–1.29	0.380	VD in Sattler’s layer	1.27	0.99–1.63	0.064	VD in Haller’s layer	1.11	0.97–1.27	0.126
**CSC vs. nAMD**															
Gender (Ref: male)	57.32	1.51–2170.33	0.029 *	Gender (Ref: male)	4.67	0.48–45.50	0.185	Gender (Ref: male)	4.45	0.47–41.90	0.192	Gender (Ref: male)	54.25	1.08–2730.27	0.046 *
Age	0.64	0.46–0.88	0.007 *	Age	0.70	0.58–0.85	<0.001 *	Age	0.67	0.54–0.84	<0.001 *	Age	0.53	0.33–0.86	0.010 *
Refractive	0.72	0.38–1.36	0.308	Refractive	1.04	0.54–2.03	0.904	Refractive	0.96	0.51–1.79	0.888	Refractive	0.76	0.35–1.63	0.479
SFCT (per 100μm)	8.16	1.30–51.14	0.025 *	VD in CC layer	0.92	0.51–1.66	0.775	VD in Sattler’s layer	1.17	0.86–1.59	0.310	VD in Haller’s layer	1.34	1.06–1.70	0.015 *

* Statistically significant: *p* ≤ 0.05; ref: reference; OR: odds ratio; CI: confidence interval; VD: vessel density; CC: choriocapillaris; PCV: polypoidal choroidal vasculopathy; nAMD: neovascular age-related macular degeneration; CSC: central serous chorioretinopathy; SFCT: subfoveal choroidal thickness.

## Data Availability

Data supporting reported results, as well as the archived datasets analyzed or generated during the study.

## Abbreviations

ANOVA: one-way analysis of variance; BCVA: best-corrected visual acuity; CC: choriocapillaris; ChT: choroidal thickness; CI: confidential interval; CSC: central serous chorioretinopathy; CVD: choroid vascular density; ETDRS: Early Treatment Diabetic Retinopathy Study Classification; FA: fluorescein angiography; ICGA: indocyanine green angiography; IQR: interquartile range; LR: likelihood ratio; nAMD: neovascular age-related macular degeneration; Non-pachy nAMD: nAMD with non-pachychoroid; Non-pachy PCV: PCV with non-pachychoroid; OCTA: Optical coherence tomography angiography; OR: odds ratio; Pachy-nAMD: nAMD with pachychoroid; Pachy-PCV: PCV with pachychoroid; PCD: pachychoroidal diseases; PCV: polypoidal choroidal vasculopathy; SFCT: subfoveal choroidal thickness; SS-OCT: swept-source optical coherence tomography; VD: vessel density; VEGF: vascular endothelial growth factor.
